# Research progress of endothelial‐mesenchymal transition in diabetic kidney disease

**DOI:** 10.1111/jcmm.17356

**Published:** 2022-05-13

**Authors:** Ying Chen, Hang Zou, Hongwei Lu, Hong Xiang, Shuhua Chen

**Affiliations:** ^1^ Department of Biochemistry and Molecular Biology School of Life Sciences Central South University Changsha China; ^2^ Center for Experimental Medical Research The Third Xiangya Hospital of Central South University Changsha China; ^3^ Department of Cardiology The Third Xiangya Hospital of Central South University Changsha China

**Keywords:** diabetic kidney disease, endothelial‐mesenchymal transition, extracellular matrix, renal fibrosis

## Abstract

Renal fibrosis is an important pathological feature of diabetic kidney disease (DKD), manifested as tubular interstitial fibrosis, tubular atrophy, glomerulosclerosis and damage to the normal structure of the kidney. Renal fibrosis can eventually develop into renal failure. A better understanding of renal fibrosis in DKD is needed due to clinical limitations of current anti‐fibrotic drugs in terms of effectiveness, cost‐effectiveness and side effects. Fibrosis is characterized by local excessive deposition of extracellular matrix, which is derived from activated myofibroblasts to increase its production or specific tissue inhibitors of metalloproteinases to reduce its degradation. In recent years, endothelial‐mesenchymal transition (EndMT) has gradually integrated into the pathogenesis of fibrosis. In animal models of diabetic kidney disease, it has been found that EndMT is involved in the formation of renal fibrosis and multiple signalling pathways such as TGF‐β signalling pathway, Wnt signalling pathway and non‐coding RNA network participate in the regulation of EndMT during fibrosis. Here, we mainly review EndMT regulation and targeted therapy of renal fibrosis in DKD.

## 1 INTRODUCTION

Diabetic kidney disease (DKD) is a serious complication of diabetes that can progress to renal fibrosis. The latter is a central step in the development of this common diabetic microvascular complication into end‐stage renal disease (ESRD).^1^ In the past ten years, the incidence and prevalence of DKD have increased significantly in China.^2^ Therefore, it is urgent to clarify the underlying mechanism of renal fibrosis in DKD and find new prevention and treatment strategies to slow down the development of diabetic renal fibrosis and avert the occurrence of ESRD.

Basically, renal fibrosis is a pathological process of excessive deposition of extracellular matrix (ECM) in the kidney tissue under the long‐term stimulation of harmful factors such as high glucose, trauma, inflammation and oxidative stress or normal wound healing disorders. Renal fibrosis can cause damage to kidney structure and function.^3^ So far, although there has been a major breakthrough in the understanding of renal fibrosis, the specific molecular mechanism of this process is still unclear, and current anti‐fibrotic drugs still have clinical limitations in terms of efficacy, cost‐effectiveness and side effects.^4^

As an important mechanism of diabetic renal fibrosis, abnormal activation of renal myofibroblasts and significant downregulation of tissue inhibitors of metalloproteinases synergistically promote the accumulation of ECM in tissues. Renal myofibroblasts are quite heterogeneous and can originate from different cell types, including resident mesenchymal cells, renal tubular epithelial cells, glomerular endothelial cells (ECs), macrophages and circulating fibroblast‐like cells derived from bone marrow stem cells. There is no doubt that epithelial/endothelial‐mesenchymal transition (EMT/EndMT) is a key step in the process of renal fibrosis.^5^, ^6^, ^7^ Since both EMT and EndMT are involved in the excessive accumulation of renal ECM, they have similar developmental processes in producing mesenchyme‐like cells. However, EndMT refers to the transition from ECs to mesenchymal cells; so, it can be considered as a special type of EMT that occurs in ECs.^8^ Under normal physiological conditions, EndMT‐producing myofibroblasts secrete ECM proteins, such as type I collagen, which together promote tissue wound repair and remodelling.^9^ Under pathological conditions, ECs in the kidney over‐secrete ECM due to persistent stimulation by inflammation and hyperglycaemia.^10^ Furthermore, downregulation of matrix metalloproteinase (MMP) activity or upregulation of tissue inhibitors of metalloproteinases (TIMPs) in the kidney leads to more ECM production and less degradation, thus promoting renal fibrosis.^11^ MMP2‐deficient mice have been reported to exacerbate renal urinary albumin excretion, accumulation of ECM in glomeruli and renal tubulointerstitial atrophy and fibrosis.^12^ It is clear that a homeostatic imbalance between ECM deposition and degradation in ECs is the primary cause of EndMT. Using three mouse models of chronic kidney disease, unilateral ureteral obstructive (UUO) nephropathy, streptozotocin (STZ)‐induced DKD and Alport nephropathy, Zeisberg et al.^7^ first tested the role of EndMT in renal fibrosis in 2008 and found that about 30%–50% of fibroblasts co‐express the endothelial marker CD31 and fibroblast markers such as α‐smooth muscle actin (α‐SMA) and fibroblast‐specific protein‐1(FSP‐1), indicating that these fibroblasts originate from endothelium cells. In this review, we will discuss the latest advances in EndMT regulation and targeted therapy in diabetic renal fibrosis.

## 2 EMT AND MMT

The EMT of renal epithelial cells has been controversial.^13^ In fact, Loeffler et al.^14^ suggested that EMT may be incomplete, meaning that complete conversion of epithelial cells to fibroblasts may be a rare event or a non‐event. However, others have confirmed the existence of at least one EMT stage.^13^ Recent studies have shown that in DKD, tripterygium glycoside attenuates EMT and apoptosis in diabetic kidney disease, by upregulating autophagy through the mTOR/Twist1 signalling pathway.^15^

Macrophages typically induce fibrosis through fibroblast recruitment, proliferation and activation. Recently, an increasing number of studies have shown that macrophages are directly involved in fibrosis through their transformation into myofibroblasts, termed macrophage‐myofibroblast transition (MMT).^16^ Co‐expression of macrophage (CD68) and myofibroblast (α‐SMA) markers was found in biopsy specimens from patients with active chronic allograft rejection, suggesting that macrophages undergo mesenchymal transformation to myofibroblasts.^17^ Moreover, approximately 50% of myofibroblasts in the kidney are CD68+/α‐SMA + double positive cells and are associated with interstitial fibrosis after chronic allograft injury,^17^ suggesting that MMT plays an essential role in renal fibrosis.

## 3 OVERVIEW OF ENDMT

Although as early as the 1970s, Markwald et al.^18^ studied the cell transformation during embryonic heart valve development and proposed that EndMT is an important pathophysiological process in embryonic growth and development, it was only recently that EndMT was found to occur in fibrotic diseases. During EndMT, ECs in fibrotic diseases gradually lose their polarity and cell adhesion substances, such as platelet endothelial cell adhesion molecule‐1, VE‐cadherin, Tie1 and Tie2, while increasing the expression of interstitial genes and proteins, such as α‐SMA, N‐cadherin, FSP‐1 and matrix metalloproteinases. Simply put, EndMT changes the polarity and normal morphology of ECs; so that, these cells lack cell‐to‐cell connections, have a high migration potential and promote the development of tissue fibrosis.^19^ In the renal microvascular environment, endothelial structures are highly heterogeneous and highly specialized from preglomerular arterioles to peritubular capillary beds.^20^ When the kidney is stimulated by injury, ECs swell to narrow the capillary gap, increase leukocyte adhesion, promote red blood cell folding, disrupt normal blood flow, reduce inward flow to lower shear stress and inhibit NO formation. All these changes plus decreased NO production and impaired vasodilation lead to decreased renal blood flow, renal ischemia, initiation of EndMT and ultimately renal fibrosis.^21^ Dysfunctional glomerular ECs can release soluble mediators, causing podocyte injury and thylakoid activation, which in turn exacerbates glomerular endothelial cell injury, creating a vicious cycle that leads to albuminuria in diabetic patients.^22^ Lovisa et al.^23^ found that inhibition of EndMT limited peritubular vascular leakage, reduced tissue hypoxia and maintained tubular epithelial cell function. Thus, EndMT is a complex process by which cells respond to stimuli.

## 4 ENDMT AND DKD

Most previous studies believe that epithelium and bone marrow are the main sources of renal fibroblasts in DKD.^24^ DKD renal fibrosis is characterized by excessive deposition of ECM,^3^ which mainly contains insoluble collagen, fibronectin, elastin, structural glycoproteins, proteoglycan and hyaluronic acid (HA).^25^ These different components play their respective roles in the kidney. Collagen molecules consist of three polypeptide chains that are intertwined to form a three‐stranded helical rope‐like collagen protofibril. They are mainly present in the endothelium, capillaries and interstitial tissues.^25^ The main function of fibronectin is to attach cells to various matrices, provide structural support and binds to the cell surface, periplasmic matrix and basement membrane.^25^ Elastin is present in tethered stalks and afferent and efferent arterioles and is responsible for the remodelling, dilation and thickening of glomerular capillary basement membrane.^25^, ^26^ Laminin is the most abundant glycoprotein in the basement membrane, and this structurally bond glycoprotein is involved in the assembly of the basement membrane by binding to cells, heparan sulphate proteoglycans and type IV collagen.^27^ Proteoglycans and hyaluronic acid are ubiquitous and present in the inner membrane. Heparan sulphate proteoglycan is an essential proteoglycan located in the glomerular basement membrane (GBM) and other membranes.^28^ Low molecular weight fucoidan ameliorates the progression of DKD in rats by maintaining GBM and glomerular structural integrity, improving glomerular filtration, protecting glycosaminoglycans from abnormal degradation and preventing the production and accumulation of advanced glycosylation end products.^29^ HA, composed of end‐to‐end stretched disaccharides, is hydrophilic and can bind large amounts of water to form a viscous hydrated gel that swells the ECM and makes it resistant to compressive forces, promoting cell migration and angiogenesis.^25^

Recent studies have shown that EndMT has emerged as a new mechanism of myofibroblast formation,^30^ which greatly contributes to the occurrence and development of renal fibrosis.^7^ Valerie et al.^31^ confirmed that EndMT is one of the important contributors of myofibroblast population in renal fibrosis. Using endothelial‐specific overexpression of Sirt3 Tg (Tie 1 Sirt3 Tg+) mice and Sirt3 knockout (SIRT3 fl/fl) mice, Srivastava et al.^32^ found that the relative fibrotic area and collagen deposition in the kidney were significantly reduced in diabetic Sirt3 Tg + mice compared with diabetic controls, while diabetic SIRT3 fl/fl mice had significantly increased fibrosis. In addition, the expression of FSP‐1 and α‐SMA in renal ECs of diabetic Sirt3 Tg + mice was significantly suppressed compared with diabetic controls, while the corresponding SIRT3 fl/fl mice exhibited higher expression levels of FSP‐1, α‐SMA and TGFβR1, suggesting that diabetic renal ECs are involved in renal fibrosis via EndMT.^32^ Lovisa et al.^23^ found that conditional knockdown of Twist1 or Snai1 in ECs reduced inflammation, inhibited EndMT and improved renal fibrosis. Overall, EndMT plays a key role in the generation of activated fibroblasts/myofibroblasts leading to fibrosis in diabetic kidney disease and is a potential therapeutic target for the treatment of DKD.

In patients with diabetic kidney disease, EndMT is a key component of renal fibrosis. Renal biopsies performed in these patients revealed marked upregulation of NOD2, downregulation of CD31 and upregulation of α‐SMA in the glomerular region of all subjects, indicating altered EC phenotype with interstitial transformation.^33^ In patients and rats with DKD, it was found that CD31 expression was decreased, α‐SMA and enolase 1 (ENO1) levels were increased and vascular endothelial damage was found. However, knocking down ENO1 reversed these responses and attenuated EndMT and the development of renal fibrosis.^34^ Furthermore, binding of lysine methyltransferase 5A to the transcription factor ETS proto‐oncogene 1 enhanced profilin 2 transcriptional activity and promoted hyperglycaemia‐induced EndMT in glomerular ECs of DKD patients and rats.^35^ These evidence supports the involvement of EndMT in the development of renal fibrosis in patients with DKD.

### 4.1 EndMT regulation

In DKD, EndMT not only causes a sharp increase in fibroblasts, but also accelerates the process of renal fibrosis.^7^ Studies have shown that many signalling pathways that regulate EndMT are involved in the process of renal fibrosis in DKD, including transforming growth factor‐β (TGF‐β) signalling pathway, Wnt signalling pathway and non‐coding RNA regulation, etc.

#### 4.1.1 TGF‐β signalling pathway

After the first report that TGF‐β1 is involved in EndMT, the TGF‐β signalling pathway has become a hot topic in EndMT research.^36^ In addition to participating in the EndMT process, TGF‐β as a growth factor is also involved in organ development, tissue repair, pro‐fibrogenic transformation and tumorigenesis.^19^, ^24^, ^37^

**FIGURE 1:**
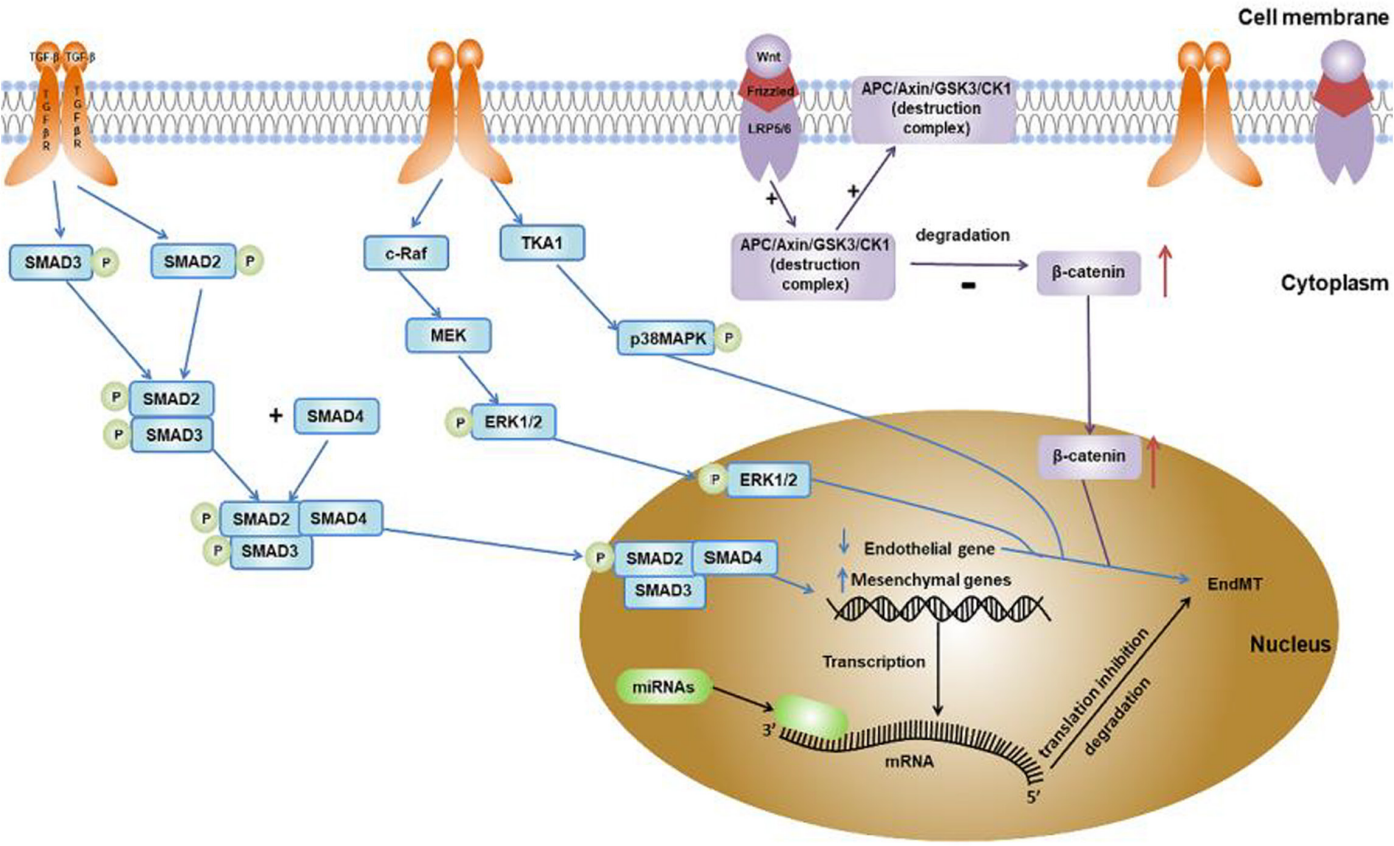
Signalling pathways of renal fibrosis mediated by endothelial‐mesenchymal transition in DKD. For example, transforming growth factor‐β (TGF‐β) signalling pathway, Wnt signalling pathway and microRNA (miRNA) network. TGF‐β, transforming growth factor‐β; TGFβR, TGF‐β receptor; miRNA and microRNA

In the process of TGF‐β‐induced EndMT, active TGF‐β first activates specific cell surface receptors with intrinsic kinase activity, thereby phosphorylating and activating Smad2/3 heterodimers. The activated Smad2/3 and cytoplasmic Smad4 form a trimer, which then enters the nucleus and induces specific target genes, including Snail, Slug and Twist, leading to the initiation of EndMT. This process is also called Smad‐dependent signalling pathway. Besides, TGF‐β can also activate other signalling pathways through its receptor complex, such as extracellular signal‐regulated kinase (ERK)/mitogen‐activated protein kinase (MAPK) pathway, p38 MAPK and phosphatidylinositol‐3‐kinase (PI3K)/AKT. Activated MAPK and AKT promote EndMT by regulating the transcription of EndMT‐related genes. This EndMT induced by TGF‐β through MAPK and AKT is called Smad‐independent signalling pathway (Figure 1).^38^, ^39^ Note that the Smad‐dependent and Smad‐independent pathways can coordinate the regulation of EndMT. For example, ERK MAPK crosstalks with the Smad‐dependent signalling pathway through phosphorylation of Smad2/3.

In DKD, TGF‐β is usually overexpressed. Therefore, by regulating the TGF‐β signalling pathways, it should be possible to perform pharmacological manipulations of renal fibrosis in DKD. For examples, the statin drug lovastatin reduces the glomerular EndMT of DKD by inhibiting oxidative stress and TGF‐β1/Smad signalling pathway, thereby improving renal function.^40^ Ergosterol is a fungal sterol that can inhibit the TGF‐β1/Smad2 signalling pathway, and ultimately reduce the proliferation of glomerular mesangial cells and ECM deposition induced by high glucose, thereby alleviating the renal fibrosis of DKD.^41^ Conversely, the increased number of circulating type 2 innate lymphocytes is partly involved in renal fibrosis through the TGF‐β1 signalling pathway, thereby aggravating DKD.^42^ It can be seen that EndMT plays a pivotal role in the occurrence and development of DKD renal fibrosis through the TGF‐β signalling pathway.

#### 4.1.2 Wnt signalling pathway

The Wnt/β‐catenin pathway is the canonical Wnt pathway, marked by the accumulation of the adhesion‐related protein β‐catenin and its translocation to the nucleus. In normal adult kidney tissue, the expression levels of Wnt/β‐catenin signalling pathway components are low, but their expression levels are rapidly upregulated in damaged kidney tissues. When the Wnt/β‐catenin pathway is activated, Wnt binds to the receptor complex composed of Frizzled and LRP5/6 and recruits the APC/Axin/GSK3/CK1 destruction complex to the cell membrane, thus avoiding the phosphorylation and degradation of β‐catenin by the destruction complex (Figure 1). Subsequently, the free β‐catenin accumulated in the cytoplasm translocates to the nucleus, regulates the expression of Wnt target genes and enhances myofibrogenesis and EndMT.^43^

It is conceivable that inhibiting the Wnt signalling pathway will slow down renal fibrosis, otherwise it will promote its occurrence. It has been proven that the traditional Chinese medicine Qishen Yiqi Dripping Pill exerts renal protection by inhibiting the Wnt/β‐catenin and TGF‐β/Smad2 signalling pathways in STZ‐induced diabetic rats. Moreover, miR‐29c inhibits Wnt/β‐catenin pathway and the formation of myofibroblasts by targeting tropomyosin 1, thereby reducing ECM production and renal fibrosis in mice.^44^, ^45^ On the contrary, miR‐27a activates Wnt/β‐catenin signalling by inhibiting the expression of secreted frizzled‐related protein‐1, thus promoting ECM deposition and renal fibrosis in DKD.^46^ Similarly, C‐reactive protein promotes EMT and renal fibrosis in STZ‐induced diabetic kidney disease by activating Wnt/β‐catenin and ERK MAPK signalling pathways, but its role in EndMT remains to be further explored.^47^ It can be said that for reducing kidney damage, it is particularly important to fully clarify the mechanism of Wnt signalling‐mediated EndMT in DKD.

#### 4.1.3 Non‐coding RNAs regulate EndMT

##### MicroRNA (miRNA) regulation

miRNAs are a group of small non‐coding regulatory RNAs approximately 21–25 nucleotides in length. They are highly conserved and tissue‐specific, and exist in a complex cellular regulatory network. miRNAs base‐pair with the complementary sequence of the 3'‐untranslated region of their target mRNAs, resulting in their target mRNA degradation and/or translation inhibition (Figure 1 and Table 1).^48^ In recent years, it has been reported that miRNAs are involved in almost all biological processes, including cell proliferation, apoptosis and differentiation.

**TABLE 1:** List of some miRNAs known to regulate endothelial cell homeostasis

Non‐coding RNAs	Mechanism	Reference
miR‐29s 和 miR‐let‐7s	By targeting working with AcSDKP	^55^
miR‐4516	By targeting miR‐4516/SIAH3/PINK1 axis	^97^
miR‐532‐5p	By targeting KRAS‐NAP1L1/P‐ERK/ETS1 axis	^98^
miR‐181b	By reducing chronic oxidative stimulation	^99^

Various miRNAs can target EMT and regulate organ fibrosis by affecting the expression of specific ligands, receptors and signalling pathways.^49^ For example, miR‐19 promotes renal fibrosis by targeting PTEN to activate the signalling pathway of Akt and induce EMT in renal tubular epithelial cells.^50^ In the kidney, miRNAs have been shown to play key regulatory roles in different stages of renal fibrosis.^48^ For example, upregulation of LncRNA‐H19 expression and downregulation of miR‐29a expression were observed in mouse models of early‐ and late‐stage diabetic renal fibrosis. After knocking out H19, miR‐29a expression was significantly increased, thereby inhibiting the expression of EndMT‐related gene FSP‐1 and reducing renal fibrosis in mice.^51^ miR‐155‐5p is another miRNA involved in the formation of renal fibrosis. miR‐155‐5p is highly expressed in the kidney tissue of UUO patients and rat models; it promotes renal fibrosis by targeting suppressor of cytokine signalling 1 (SOCS1) and SOCS6 and then increasing the phosphorylation and activation of the transcription factor STAT3.^52^ Significant downregulation of miR‐126‐3p was observed fibrotic kidney tissues from patients, and overexpression of miR‐126‐3p in ECs partially blocked EndMT and slowed the progression of renal fibrosis.^53^ Since one miRNA can target multiple genes simultaneously, and multiple miRNAs can coordinately regulate a single mRNA, there is extensive crosstalk in miRNA regulation of mRNAs. For example, the cross‐regulation between miR‐29 and let‐7 enhances the anti‐fibrotic peptide N‐acetylseryl‐aspartyl‐lysyl‐proline(AcSDKP), improves renal fibrosis and protects the kidneys.^54^ Treatment of cultured cells with an angiotensin converting enzyme inhibitor (ACEi) or in combination with the anti‐fibrotic peptide AcSDKP prevented TGF‐β‐induced downregulation of miR‐29 and miR‐let‐7 in ECs, inhibited EndMT and impeded renal fibrosis progression.^55^ It is now clear that miRNAs are new and attractive targets for the treatment of renal fibrosis and DKD. However, incorporating miRNA‐based therapies into clinical trials remains a major challenge, and more research is needed to address issues such as off‐target and multiple‐target effects.

##### Circular RNA (circRNA) regulates EndMT

Exonic circRNAs are another subclass of non‐coding RNAs, which are circularized by back‐splicing and function as miRNA sponges, RNA splicing factors and protein scaffolds to regulate mRNA transcription and expression.^56^ circRNA are active in regulating EndMT. For example, circHECW2 acts as a miR‐30d sponge to increase autophagy‐related 5 expression and activate Notch1 pathway‐induced pathological EndMT.^57^ Peng et al.^58^ found that circRNA increased ECM accumulation in kidneys of db/db mice by downregulating the ion channel TRPC1, a target protein of miR‐135a, and promoted proteinuria and renal fibrosis. It is speculated that circRNAs may be another important target of non‐coding RNAs in regulating EndMT and renal fibrosis. Although there are few related studies, the understanding of the role of circRNAs in EndMT is just beginning.

##### Long non‐coding RNA (lncRNA) regulates EndMT

lncRNAs are non‐coding RNAs over 200 nucleotides in length. lncRNAs regulate gene expression through multiple mechanisms, such as directly affecting transcription, regulating chromatin modification complexes, regulating mRNA processing and stability and acting as miRNA sponges.^59^ A study shows that the lncRNA GATA6‐AS promotes hypoxia‐induced EndMT in human umbilical vein ECs through histone methylation.^60^ It was also found that knockdown of lncRNA MALAT1 downregulated Snail expression, thus improving EndMT in vascular ECs.^61^ In addition, a few studies have reported extensive crosstalk between lncRNAs and miRNAs. For example, Xiang et al.^62^ reported that lncRNA MALAT1 competitively bound miR‐145 and directly targets TGF‐βR2 and SMAD3 to inhibit TGF‐β1 induced EndMT. In mice, lncRNA Erbb4‐IR promotes the progression of DKD renal fibrosis by repressing miR‐29b transcription and increasing collagen I and IV expression levels.^63^ All these data clearly suggest that lncRNAs mediate the development of EndMT associated renal fibrosis.

#### 4.1.4 Hedgehog signalling pathway

**FIGURE 2:**
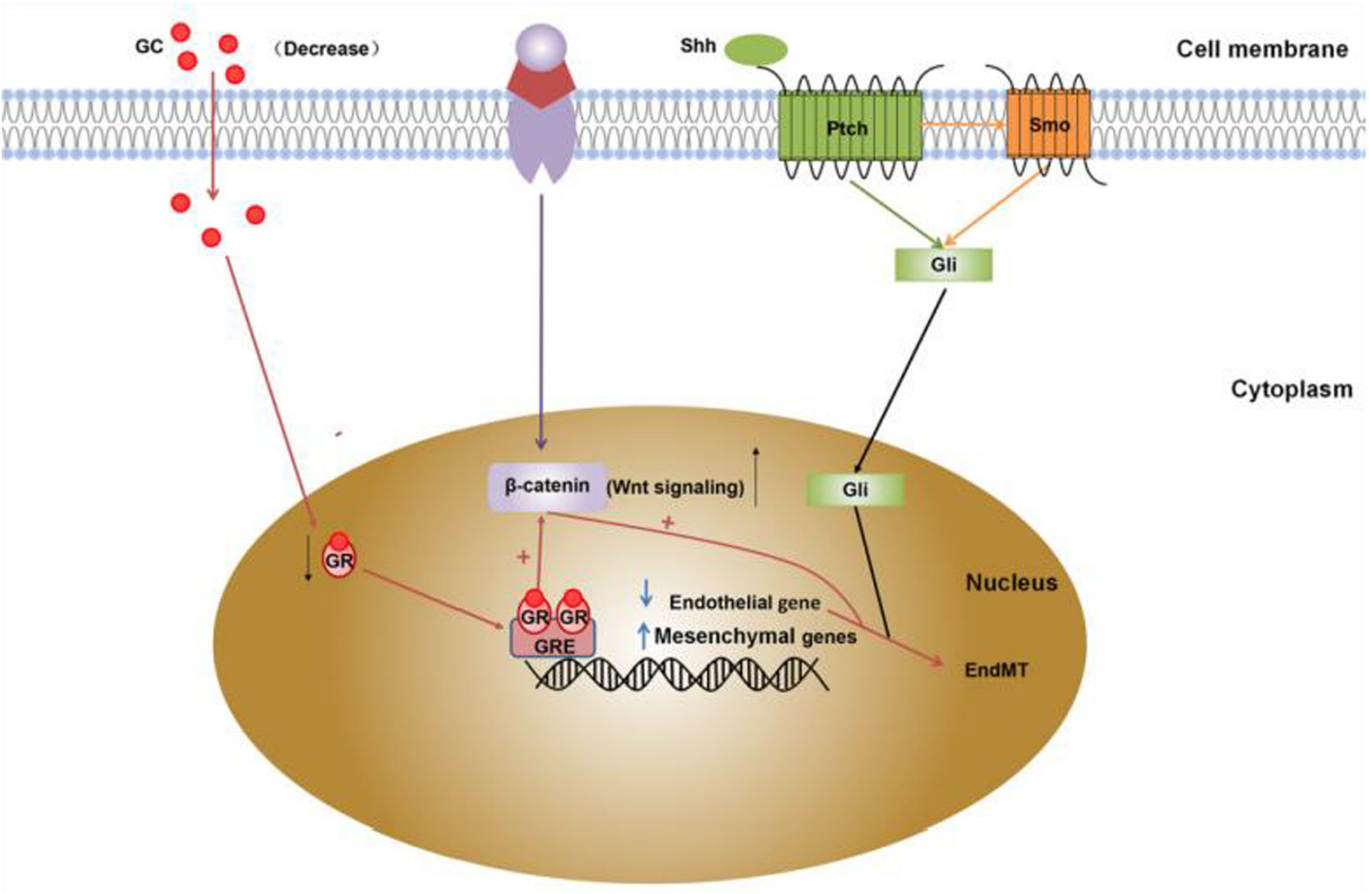
Signalling pathways of renal fibrosis mediated by endothelial‐mesenchymal transition in DKD, including Hedgehog signalling pathway and glucocorticoid receptor signalling pathway. GRE, glucocorticoid response element; Shh, Sonic Hedgehog; GC, glucocorticoid; GR, glucocorticoid receptor; Ptch, patched; Smo, smoothened

The Hedgehog (HH) pathway includes three ligands: Sonic Hedgehog (Shh), Indian Hedgehog (IHH) and Desert Hedgehog (DHH), which are lipid‐modified proteins that can act in autocrine and paracrine manners.^64^, ^65^, ^66^ The HH pathway consists of two transmembrane proteins, Patched (Ptch) and Smoothened (Smo), which act as signal transducers similar to G protein‐coupled receptors (Figure 2).^67^ A study of renal disease showed that the expression of hedgehog‐interacting protein (HHIP) was significantly elevated in the kidney of diabetic mice, enhancement of HHIP promoted endothelial EndMT and apoptosis, while knockdown of HHIP significantly ameliorated renal injury.^68^ In addition, the HH pathway may promote renal fibrosis by cooperating with other signalling pathways. Hyperglycaemia enhances ROS production and stimulates renal HHIP gene expression by activating NADPH oxidase 4. Elevated HHIP activates the TGF‐β1‐Smad2/3 cascade and promotes EndMT associated with renal ECs.^69^ It is evident that HH is an important signalling pathway for the development of renal fibrosis caused by EndMT‐causing; so, blocking this pathway can reduce renal fibrosis and achieve renal protection.

#### 4.1.5 Glucocorticoid receptor signalling pathway

Glucocorticoid receptor (GR) is a nuclear hormone receptor that is important for the maintenance of health and disease progression. GRs mediate the biological effects of steroid hormones in various tissues, including the kidney (Figure 2).^70^ For example, glucocorticoids enhance the production of TGF‐β‐induced plasminogen activator inhibitor‐1 in human proximal tubular cells by binding to GR, thereby inhibiting ECM degradation and promoting renal fibrosis.^71^ In DKD, endothelial GR knockout diabetic mice displayed higher relative area of fibrosis and collagen deposition, significantly upregulated α‐SMA, increased active β‐catenin levels and significant CD31 downregulation in renal ECs compared with diabetic control mice, suggesting that GR deficiency mediates EndMT in ECs in diabetic kidney disease by upregulating Wnt signalling, ultimately promoting the progression of renal fibrosis and glomerulosclerosis.^72^ Therefore, endothelial GR is a key molecule involved in regulating the process of renal fibrosis and may be a new target for exploring the prevention of renal fibrosis in DKD.

#### 4.1.6 FGFR1 signalling pathway

Fibroblast growth factor receptors (FGFRs) are a family of four tyrosine kinase receptors that can be specifically activated by cognate ligands.^73^ FGFRs are involved in various biological processes such as cell proliferation, migration and EndMT.^74^, ^75^ FGFR1, a key anti‐EndMT molecule, is an endothelial receptor required for counteracting EndMT.^76^ Chen et al.^77^ reported that endothelial FGFR1 deficiency was associated with increased TGF‐β signalling and Smad2 activation, which exacerbates EndMT through enhanced TGF‐β signalling. However, inhibition of FGFR1 signalling reduces EndMT in ECs. Further, Terzuoli et al.^78^ found that pharmacological inhibition of FGFR1 by AZD4547 blocked the formation of the FGFR1/TRAF6 complex, hence inhibiting TRAF6/NF‐κB inflammatory signalling and attenuating renal inflammatory injury and renal dysfunction. It has also been reported that diabetic FGFR1 knockout mice have significantly higher EndMT and EMT levels than diabetic control mice.^79^ Thus, FGFR1 signalling may play a key role in preventing endothelial dysfunction and regulating EndMT and DKD renal fibrosis. Clinically relevant studies on the mechanisms of negative regulation of FGFR may help to find new ways to prevent renal dysfunction and fibrosis.

#### 4.1.7 SIRT3 signalling pathway

Sirtuin3 (SIRT3), an important member of the sirtuin family, is a major deacetylase located in the mitochondrial matrix.^80^ Chung et al.^81^ showed that a correlation exists between mitochondrial injury, inflammation and renal fibrosis, and that mitochondrial metabolism is critical for renal cell homeostasis. SIRT3, a key regulator of nematocytes, has been increasingly focused on SIRT3 in the fight against renal fibrosis.^82^ Lin et al.^83^ showed that SIRT3 knockout in mice resulted in increased levels of EndMT and ROS, promoting renal dysfunction, while in SIRT3 knock‐in EC specific transgenic mice, renal fibrosis and EndMT as well as oxidative stress were ameliorated. Endothelial SIRT3 regulates glucose and lipid metabolism and related EndMT processes by maintaining control of TGF‐β/Smad3 signalling in the kidney of diabetic mice.^32^ In addition, SIRT3‐deficient EndMT induces mesenchymal transformation of renal epithelial cells and activates fibrotic responses throughout the glycolytic kidney.^32^ Thus, SIRT3 may be the key signalling pathway that inhibits EndMT.

#### 4.1.8 Other mechanisms affecting EndMT

Fatty acid oxidation (FAO) has recently been found to be an important factor in regulating EndMT. Under physiological conditions, FAO metabolism in ECs is active to ensure the normal level of acetyl CoA, an EndMT sensor, and maintain the stability of Smad7. After TGF‐β activated EndMT, the expression of FAO‐related genes, FAO metabolism, acetyl CoA synthesis and Smad7 acetylation were all reduced, thereby weakening the inhibition of EndMT, promoting endothelial fibrosis and ultimately impairing renal function.^84^ Recent studies have shown that Sirtuin 3 (SIRT3) is involved in the FAO regulation of cisplatin‐induced acute kidney injury. When SIRT3 was knocked out, FAO dysfunction was aggravated; conversely, when the expression of SIRT3 was upregulated, FAO disorder was alleviated and renal function was improved, indicating that FAO has a protective effect on renal function.^85^ In addition to FAO, glycolysis is the main energy supplier to ECs, accounting for approximately 75%–85% of the total ATP production.^86^ When subjected to stimulation by pro‐inflammatory factors, the glycolytic flux of ECs increases, which contributes to the proliferation and migration of ECs and may promote the EndMT process ECs.^87^ Srivastava et al.^32^ found that overexpression of SIRT3 inhibited α‐SMA as well as glycolytic enzymes such as hexokinase, hexokinase in renal ECs in mice with diabetic kidney disease phosphofructokinase activity and lactate levels, thereby inhibiting EndMT and protecting the kidney. Nevertheless, so far, there are few reports on the regulatory role of FAO and glycolysis in EndMT‐mediated diabetic renal fibrosis; so, there is still a long way to go for anti‐fibrotic drug treatment research.

Chronic irritation and inflammation are also one of the factors that induce EndMT.^88^ According to reports, inflammatory cytokines TNF‐α and IL‐1β can trigger EndMT.^88^ The release of excessive inflammatory mediators will promote the occurrence and development of renal fibrosis in DKD from many aspects such as fibroblast activation and EMT/EndMT.^89^ Mechanistically, the activation of NOD‐like receptor protein 3 (NLRP3) inflammasomes and subsequent release of IL‐1β can promote UUO‐induced renal fibrosis, but gemigliptin, an anti‐hyperglycaemic agent, inhibits the activation of NLRP3 inflammasomes induced by TGF‐β/NF‐κB signalling, thereby downregulating fibrosis genes and protecting renal function.^90^ Hence, it is possible to control or reverse the occurrence of renal fibrosis by inhibiting the activation of inflammasomes or the excessive release of IL‐1β and the later amplification of inflammatory signals.

### 4.2 EndMT targeted therapy

With the in‐depth study of renal fibrosis, it has gradually revealed the indispensable role of EndMT in promoting renal fibrosis. Accordingly, research on anti‐kidney fibrosis drugs with EndMT as the intervention target has gradually emerged. Dipeptidyl peptidase‐4 (DPP‐4) inhibitors, such as gemigliptin, sitagliptin and liraglutide, first entered the market as anti‐diabetic drugs. Later, sitagliptin and liraglutide were found to reduce renal cell apoptosis by increasing the protein expression of survival factor GRP78. Furthermore, these DPP‐4 inhibitors can inhibit the phenotypic transformation of renal microvascular smooth muscle cells and inhibit EndMT by upregulating bone morphogenetic protein receptor 2 and downregulating TGF‐β1, thus reducing renal fibrosis.^91^ Lovastatin can significantly inhibit high glucose‐induced glomerular endothelial cell EndMT and TGF‐β1 signalling, thereby slowing down the progression of renal fibrosis.^44^ Although there is no previous report of lovastatin inhibiting EndMT to alleviate renal fibrosis caused by hyperglycaemia, its effect has only been validated in animal experiments and has not yet entered the clinical trial stage. AcSDKP has been reported to exert anti‐renal fibrosis effects by inhibiting DPP‐4 levels and restoring miRNA crosstalk between miR‐29 and miR‐let‐7.^92^ Therefore, we believe that AcSDKP may be an effective therapeutic target for DPP‐4‐related pathways in diabetic renal fibrosis. So far, DPP‐4 targeted therapy seems to be a promising approach for the treatment of kidney disease. However, due to the different mechanism and metabolism of each drug, it is unclear whether other DPP‐4 inhibitors can effectively inhibit the process of renal fibrosis.

In addition to DPP‐4 inhibitors, liraglutide, empagliflozin, SIRT3 agonists, ROCK inhibitors, glycolysis inhibitors, ACEis and AcSDKP all targetedly inhibit EndMT and show anti‐fibrotic therapeutic effect.

Liraglutide attenuates diabetes‐associated EndMT via the AMPK pathway.^93^ Empagliflozin, a novel selective sodium‐glucose co‐transport protein 2 inhibitor, inhibits EMT in proximal renal tubules and EndMT in peritubular capillaries and slows the progression of renal fibrosis in diabetic mice.^94^ SIRT3 has long been an important molecular target against renal fibrosis. Increased SIRT3 expression is beneficial in reducing renal EndMT and renal fibrosis caused by accumulation of EndMT‐derived myofibroblasts.^32^ ROCK inhibitors are effective in preventing the progression of DKD.^95^ Activation of ROCK1 by high glucose induces glomerular endothelial EndMT, increases EC permeability and increases renal fibrosis, whereas fasudil treatment inhibits glomerular EndMT mice and attenuates renal injury in early DKD.^96^ Glycolysis inhibitors are effective in reducing renal fibrosis and EndMT. Renal injury in DKD activates Twist and Snail, leading to vascular rupture, which triggers glycolysis and impairs renal function; however, silencing Twist or Snail in ECs can inhibit glycolysis to block EndMT.^33^ In recent years, ACEis and AcSDKP have been gradually studied for anti‐EndMT. A new study revealed that ACEi in combination with AcSDKP counteracts EndMT in the kidneys of diabetic mice and prevents renal fibrosis.^55^

It can be seen that these drugs or inhibitors all have a certain effect in reducing EndMT, and can be used as a combination therapy or precise targeted therapy for EndMT and renal fibrosis. Although the effects of these drugs or inhibitors on EndMT are credible, some of them have only been observed in animal models and have not yet entered clinical trials; so, more preclinical and clinical trials are still needed to verify their feasibility.

## 5 CONCLUSIONS

In summary, the occurrence of EndMT is due to the joint action of certain cytokines from multiple signal pathways, which drive the formation of renal fibrosis in DKD. However, on the one hand, the understanding of EndMT‐mediated renal fibrosis in DKD has just begun, and its related regulation mechanism is still unclear. On the other hand, although we have a preliminary understanding of how EndMT works at the cellular or animal level, further clinical trials are needed to confirm and verify. In the future, the focus should be to deeply explore the role of EndMT in renal diseases, and strive to find potential new targets for the treatment of renal fibrotic diseases, which is of great significance for the development of anti‐fibrosis drugs.

## AUTHOR CONTRIBUTIONS

Ying Chen: Conceptualization (equal); Resources (lead); Software (lead); Writing, original draft (lead); Writing, review & editing (equal). Hang Zou: Resources (supporting); Software (equal); Visualization (equal). Hongwei Lu: Funding acquisition (equal); Resources (supporting); Visualization (equal). Hong Xiang: Resources (supporting); Visualization (equal). Shuhua Chen: Funding acquisition (lead); Resources (supporting); Supervision (lead); Writing, review & editing (equal).

## CONFLICT OF INTEREST

The authors declare that they have no conflict of interest.

## CONSENT FOR PUBLICATION

All authors agree on the final version of the manuscript.

## Data Availability

The review is exempt from data sharing.
